# Identification of potential functions of polo-like kinase 1 in male reproductive development of the oriental river prawn (*Macrobrachium nipponense*) by RNA interference analysis

**DOI:** 10.3389/fendo.2022.1084802

**Published:** 2022-12-05

**Authors:** Shubo Jin, Wenyi Zhang, Pengchao Wang, Sufei Jiang, Hui Qiao, Yongsheng Gong, Yan Wu, Yiwei Xiong, Hongtuo Fu

**Affiliations:** ^1^ Key Laboratory of Freshwater Fisheries and Germplasm Resources Utilization, Ministry of Agriculture and Rural Affairs, Freshwater Fisheries Research Center, Chinese Academy of Fishery Sciences, Wuxi, China; ^2^ Wuxi Fisheries College, Nanjing Agricultural University, Wuxi, China

**Keywords:** *Macrobrachium nipponense*, polo-like kinase 1, RNAi analysis, histological observations, testis development

## Abstract

Polo-like kinase 1 (*Plk1*) has multiple functions in the cell cycle, including in the maturation of centrosomes during the G2/M transition, the separation of centrosomes, and the activation of cyclin-dependent kinase 1 expression and spindle assembly. In this study, we investigated the potential regulatory roles of *Plk1* in the reproductive development of the male oriental river prawn (*Machrobrachium nipponense*). The full cDNA sequence of *Mn*-*Plk1* was 2360 base pairs long, with an open reading frame of 1836 base pairs encoding 611 amino acids. Protein sequence alignment identified a conserved serine/threonine kinase domain and two Polo-boxes. Phylogenetic tree analysis revealed that *Mn*-*Plk1* had the closest evolutionary distance with *Plk1*s of freshwater prawns and then with those of crustacean species, whereas the evolutionary distance with mollusks was much more distant. Quantitative PCR analysis predicted that *Mn*-*Plk1* plays essential roles in the regulation of gonad development. RNA interference analysis and histological observations showed that expression of insulin-like androgenic gland hormone decreased as the expression of *Mn-Plk1* decreased, and fewer than 5% of cells were sperm cells at day 14 in the *dsPlk1* injected prawns. This result indicated that *Plk1* positively regulated testis development in *M. nipponense* by affecting the expression of this hormone. Our results highlight the functions of *Plk1* in *M*. *nipponense* and provide valuable information that can be applied to establish artificial techniques to regulate testis development in this species.

## Introduction

The X-organ–SG complex (XO–SG) is a principal neuroendocrine gland in crustaceans, and it is located in the eyestalk. It stores and releases many neurosecretory hormones (1) that play essential roles in the regulation of many biological functions in crustacean species (2–12). The neurosecretory hormones secreted by the XO–SG have been reported to have negatively regulatory effects on both testis and ovarian development of the oriental river prawn (*Macrobrachium nipponense*) (13–15). For example, Qiao et al. (2015) showed that knockdown of the expression of the gonad-inhibiting hormone gene from female *M. nipponense* promoted ovarian development, as verified by RNA interference (RNAi) (13). Testis development was induced by stimulating the expression of the insulin-like androgenic gland hormone (*IAG*) gene in *M. nipponense* when eyestalks were ablated in male prawns (14, 15). *IAG* is a well-studied gene among crustacean species. It promotes male sex differentiation and the establishment of the male sex characteristics, especially testis development (16, 17). In *Macrobrachium rosenbergii*, decreased *IAG* expression caused by RNAi inhibited male sex differentiation and development of secondary sexual characteristics, especially spermatogenesis (18). Similar functions of *IAG* are well known in many other crustacean species (19–23).

Researchers have conducted transcriptome profiling analysis of the androgenic gland after the ablation of eyestalks from male *M. nipponense* in order to identify metabolic pathways and genes that are involved in the reproductive development of male *M. nipponense* (14). They proposed that the cell cycle plays essential roles in the regulation of male reproductive development in *M. nipponense* because it was the main metabolic pathway enriched in differentially expressed genes (14). The cell cycle ensures correct cell proliferation and plays crucial regulatory roles in the prevention and/or correction of damaged DNA, mutations, and genetic abnormalities (24, 25). Thus, the up-regulated and enriched genes in the metabolic pathway of the cell cycle were predicted to regulate the process of reproductive development in male *M. nipponense* based on the negative relationship between the neurosecretory hormones secreted by the XO–SG and reproductive development of male prawns.

Previously, polo-like kinase 1 (*Plk1*) was found to be enriched in the metabolic pathway of the cell cycle, and its expression was significantly up-regulated in the androgenic gland after eyestalk ablation in male *M. nipponense* (14). This finding suggested that *Plk1* may promote the reproductive development of male *M. nipponense*. The cell cycle process is regulated by a variety of kinases, including those in the *Plk* family. *Plk1* was the first and continues to be the most studied *Plk*, and it is involved in regulation of the cell cycle. It can promote the maturation of centrosomes during the G2/M transition and the separation of centrosomes, and it can regulate the activation of cyclin-dependent kinase 1 and spindle assembly (26, 27). *Plk1* proteins are reported to be expressed during the late G2/M phase, regulating the process of mitosis in all animal species (28). Previous studies have shown that *Plk1* plays essential roles in therapies designed to treat a variety of cancers, including primary colorectal cancer (29), prostate and pancreatic cancers (30), and rectal cancer (31). However, studies of the effects of *Plk1* on gonad development are rare. Knockdown of the expression of *Plk1* was shown to inhibit female meiosis in the fruit fly Drosophila melanogaster, indicating that *Plk1* is involved in the process of oogenesis (32). Additionally, the *Plk1* of the blood fluke Schistosoma mansoni was reported to be a key mitotic kinase that is involved in the process of parasite reproduction (33).

*M. nipponense* is widely distributed in freshwater and low-salinity estuarine regions in China and other Asian countries. It is a commercially important species in China, providing huge economic benefits (34). The rapid gonad development of hatchlings has restricted the sustainable development of the *M. nipponense* industry, as newly hatched male and female *M. nipponense* can reach sexual maturity within 40 days after hatching during the reproductive season (35). Rapid gonad development affects the market size of *M. nipponense* because it has negative regulatory effects on growth performance (36, 37). Thus, an artificial technique to regulate the process of gonad development in this species is urgently needed.

The goal of this study was to investigate the potential functions of *Plk1* in male reproductive development of *M*. *nipponense* using quantitative real-time PCR (qPCR), RNAi, and histological observations. Our results can be applied to development of an artificial technique to regulate the process of gonad development in *M. nipponense*. They also provide a basis for further studies of the mechanism of male reproductive development in other crustacean species.

## Methods and materials

### Ethics approval

Permission to conduct the experiments was obtained from the the Freshwater Fisheries Research Center (2021JBFM02, 12-06-2021). All experiments were performed in accordance with Jiangsu Laboratory’s Animal Management Guidelines (014000319/2008-00079). All *M. nipponense* used in this study were collected from the Dapu *M. nipponense* Breeding Base (120°13′44″E, 31°28′ 22″N; Wuxi, China). All prawns were maintained for 3 days in aerated freshwater with dissolved oxygen content ≥ 6 mg/L prior to tissue collection. An ice bath was used to anesthetize the prawns when tissues were collected (38).

### Tissue collection

Testis were collected for the synthesis of templates and used for rapid amplification of cDNA ends (RACE) cloning. Different tissues collected for qPCR analysis included eyestalks, brain, heart, hepatopancreas, muscle, gonad, and gills from both male (body weight of 2.87-4.12 g) and female (body weight of 1.84-2.12 g) *M. nipponense*. Specimens at different developmental stages were collected from a full-sib population every 5 days during their maturation process. The body weights of the full-sib population were from 0.003-0.021 g. Testis and androgenic gland were sampled during both the reproductive season in July (water temperature of 30 ± 2°C, illumination time ≥ 16 h) (body weight of 3.12-4.47 g) and the non-reproductive season in January (water temperature of 13 ± 2°C, illumination time ≤ 12 h) (body weight of 2.15-3.97 g). For each tissue, sex, and time point, five samples were pooled together to form a biological replicate, and three biological replicates were used for qPCR analysis. All collected tissues were immediately preserved in liquid nitrogen until used for qPCR analysis.

### RACE

**Table 1** lists the specific primers used for *Mn-Plk1* cloning. The primers were designed using the Primer-BLAST tool from NCBI (http://www.ncbi.nlm.nih.gov/tools/primer-blast/) based on the open reading frame (ORF) of *Mn-Plk1*. The full-length cDNA of *Mn-Plk1* was cloned using the RACE technique following the methods described in previous studies (39, 40). Briefly, the total RNA from the testis was extracted using RNAiso Plus Reagent (Takara, Shiga, Japan), and the extracted total RNA was then used to synthesize the templates for 3′cDNA and 5′cDNA cloning using the 3′-Full RACE Core Set Ver.2.0 kit and the 5′-Full RACE kit (Takara), respectively. After the assembling, two pairs of primer were designed based on the full-length cDNA sequence of *Mn-Plk1*, in order to verify the accuracy of *Mn-Plk1* cDNA sequence (**Table 1**).

**Table 1:** Universal and specific primers used in this study.

Primer name	Nucleotide Sequence(5′→3′)	Purpose
PLK-3GSP1	CACTATCACCAGCTGAAGAAT	FWD first primer for PLK 3′ RACE
PLK -3GSP2	AACTCTTAAGCTCTCAAGCAT	FWD second primer for PLK 3′ RACE
PLK -5GSP1	CTTTTTACATAATATTTCTGGG	RVS first primer for PLK 5′ RACE
PLK -5GSP2	AACCCATGAAAGCCGACAACAT	RVS second primer for PLK 5′ RACE
3′RACE OUT	TACCGTCGTTCCACTAGTGATTT	RVS first primer for 3′ RACE
3′RACE IN	CGCGGATCCTCCACTAGTGATTTCACTATAGG	RVS second primer for 3′ RACE
5′RACE OUT	CATGGCTACATGCTGACAGCCTA	FWD first primer for 5′ RACE
5′RACE IN	CGCGGATCCACAGCCTACTGATGATCAGTCGATG	FWD second primer for 5′ RACE
PLK-F1	GTTATTTTTCGGCTGTTTTGGG	Primers for cDNA sequence verification
PLK-R1	GAATGTTGTCTCCATCGGCAAG	
PLK-F2	TCAATGAAGATGAGTGTGAGGA	
PLK-R2	AGTTATGTCTACATAAGACTAC	
PLK RNAi-F	TAATACGACTCACTATAGGGCCATCTGAACAGCAAGCAAA	FWD primer for RNAi analysis
PLK RNAi-R	TAATACGACTCACTATAGGGACACCAATGGAGTCGTCACA	RVS primer for RNAi analysis

### Structural analysis of *Mn-Plk1*

The structural characteristics of *Mn- Plk1* were analysed using the BLASTx and BLASTN search program (http://www.ncbi.nlm.nih.gov/BLAST/) and the ORF Finder tool (http://www.ncbi.nlm.nih.gov/gorf/gorf.html). The theoretical isoelectric point and the molecular weight of *Mn-Plk1* protein were measured using the ComputepI/Mwtool software (http://ca.expasy.org/tools/pi_tool.html). **Table 2** lists the accession numbers of amino acid sequences of different species, which were used to construct the phylogenetic tree of *Plk1* using MEGA X, the maximum-likelihood method, and the bootstrap method with 1000 replications.

**Table 2:** Sequences used in this study.

Species	Accession number
*Macrobrachium nipponense*	
*Procambarus clarkii*	XP_045623220.1
*Penaeus monodon*	XP_037785985.1
*Penaeus japonicus*	XP_042864001.1
*Penaeus vannamei*	XP_027211725.1
*Portunus trituberculatus*	XP_045137394.1
*Pollicipes pollicipes*	XP_037092997.1
*Amphibalanus amphitrite*	XP_043236979.1
*Crassostrea virginica*	XP_022292480.1
*Crassostrea gigas*	XP_011446350.1
*Mytilus coruscus*	CAC5390214.1
*Pomacea canaliculata*	XP_025088006.1
*Mercenaria mercenaria*	XP_045175493.1

### qPCR analysis

qPCR was used to determine the relative mRNA expression of *Mn-Plk1* in the different tissues. **Table 1** lists the primers used for qPCR analysis, which was performed following previously described methods (39, 40). Briefly, RNAiso Plus Reagent was used to extract the total RNA from each tissue following the manufacturer’s protocol. After removing genomic DNA by gDNA Eraser, the total RNA was used to synthesize the cDNA template using PrimeScript™ RT reagent Kit (Takara) based on the manufacturer’s protocol. The UltraSYBR Mixture (CWBIO, Beijing, China) with the SYBR Green RT-qPCR assay was used to determine the expression level of *Mn-Plk1* in each tissue, and the Bio-Rad iCycler iQ5 Real-Time PCR System (Bio-Rad, Hercules, CA, USA) was used to perform the qPCR analyses. The amplification protocols for qPCR analysis were 95°C for 10 min, followed by 37 cycles of 95°C for 10 s and 60°C for 1 min. All primers for qPCR analysis were listed in **Table 3**. The eukaryotic translation initiation factor 5A is a stable reference gene for qPCR analysis in *M. nipponense*, and it was used in the present study (41). The relative mRNA expression levels of *Mn-Plk1* were calculated using on the 2^−ΔΔCT^ comparative CT method (42).

**Table 3:** q PCR primers used in this study.

Primer name	Nucleotide Sequence(5′→3′)	TM	Amplification efficiency	Product size (bp)
PLK -RTF	CATGGGGGCTGTTACATACATTG	65.6°C	98.3%	153
PLK -RTR	GTTGAAGTCTTGACGCACATCAG	65.3°C		
IAG-RTF	CTGACCACACCTACTGAAGACAA	64.7°C	97.9%	127
IAG-RTR	CGTTTTCGATAAGAGGTCAAGCC	64.9°C		
EIF-F	CATGGATGTACCTGTGGTGAAAC	65.2°C	98.9%	139
EIF-R	CTGTCAGCAGAAGGTCCTCATTA	65.1°C		

### RNAi analysis

The potential function of *Plk1* in reproductive development of male *M. nipponense* was investigated by RNAi, which has been widely used in gene functional analysis in this species. **Table 1** lists the specific RNAi primer with a T7 promoter site, which was designed using Snap Dragon tools (http://www.flyrnai.org/cgibin/RNAifind_primers.pl) based on the ORF of *Mn-Plk1*. The *Mn-Plk1* dsRNA (*dsPlk1*) was synthesized using the Transcript Aid™ T7 High Yield Transcription kit (Fermentas, Waltham, MA, USA) following the manufacturer’s protocol, and the green fluorescent protein dsRNA (*dsGFP*) was also synthesized and used as the negative control (43).

Six hundred male *M*. *nipponense* were collected from the Dapu *M. nipponense* Breeding Base and randomly divided into the *dsPlk1* group (RNAi) and *dsGFP* group (control) (N = 300). These prawns were collected at approximately 5 months after hatching and had a body weight of 3.14−4.32 g. The *dsPlk1* and *dsGFP* were injected into the prawns through the cardio coelom with the injected dose of 4 μg/g according to previously described methods (44). *dsPlk1* and *dsGFP* were injected once and then again with the same dose after 7 days. Androgenic gland samples (N=15) were collected from both the control group and the RNAi group on days 1, 7, and 14 after *dsGFP* or *dsPlk1* injection. The *Mn-Plk1* mRNA expression levels were measured by qPCR to confirm the silencing efficiency. The mRNA expression of *Mn-IAG* was also measured using the same cDNA templates to analyse the regulatory relationship between *Plk1* and *IAG* in *M. nipponense*.

### Haematoxylin and eosin (HE) staining

The morphological differences between testis samples taken from the *dsPlk1*- and *dsGFP-*injected prawns were assessed by histological observations of tissues stained with HE. The histological observations were performed following previously described methods (45, 46). The slides were observed under an Olympus SZX16 microscope (Olympus Corporation, Tokyo, Japan). The cell types were labelled according to the previous studies (14, 15), and the numbers of each cell type were counted through the whole testis after the treatment of *dsGFP* and *dsPlk1*.

### Statistical analysis

SPSS Statistics 23.0 (IBM, Armonk, NY, USA) was used to evaluate differences in *Mn-Plk1* expression levels, followed the independent samples t-test and analysis of ANOVA variance (least significant difference and Duncan’s multiple range tests). Quantitative data were expressed as mean ± standard deviation. *P* < 0.05 and *P* < 0.01 were considered to be differentially expressed and significantly differentially expressed, respectively.

## Results

### Sequence analysis

**Figure 1:**
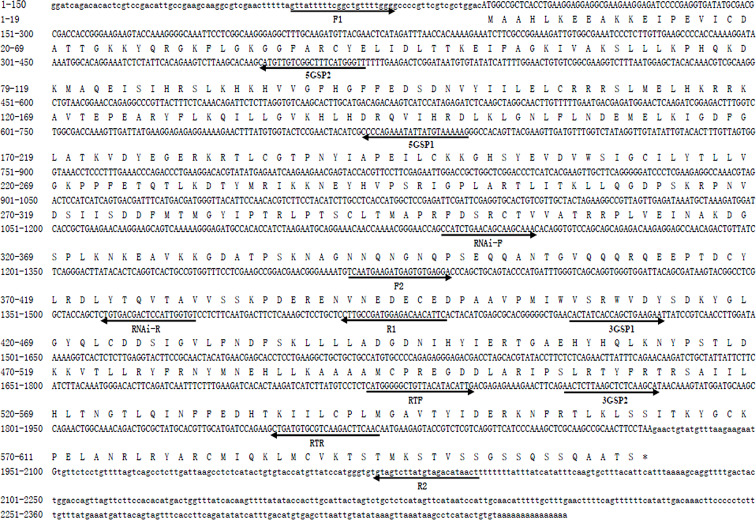
Nucleotide and deduced amino acid sequence of *Mn-Plk1*. The nucleotide sequence is displayed in the 5′–3′ directions and numbered at the left. The deduced amino acid sequence is shown in a single capital letter amino acid code. Arrows indicated the location and direction of each primer. 3′ UTR and 5′ UTR are shown with lowercase letters. Codons are numbered at the left with the methionine (ATG) initiation codon, an asterisk denotes the termination codon (TGA).

The full-length cDNA sequence of *Mn-Plk1* was 2,360 base pairs (bp) long, with a 5′ and a 3′ untranslated region of 92 bp and 432 bp, respectively. The ORF of *Mn-Plk1* was 1,836 bp long and encoded 611 amino acids (**Figure 1**). The cDNA sequence of *Mn-Plk1* was submitted to NCBI with the accession number OP379748. The theoretical isoelectric point of *Mn-Plk1* was 4.90, and the molecular weight of the protein was 18.466 kDa.

**Figure 2:**
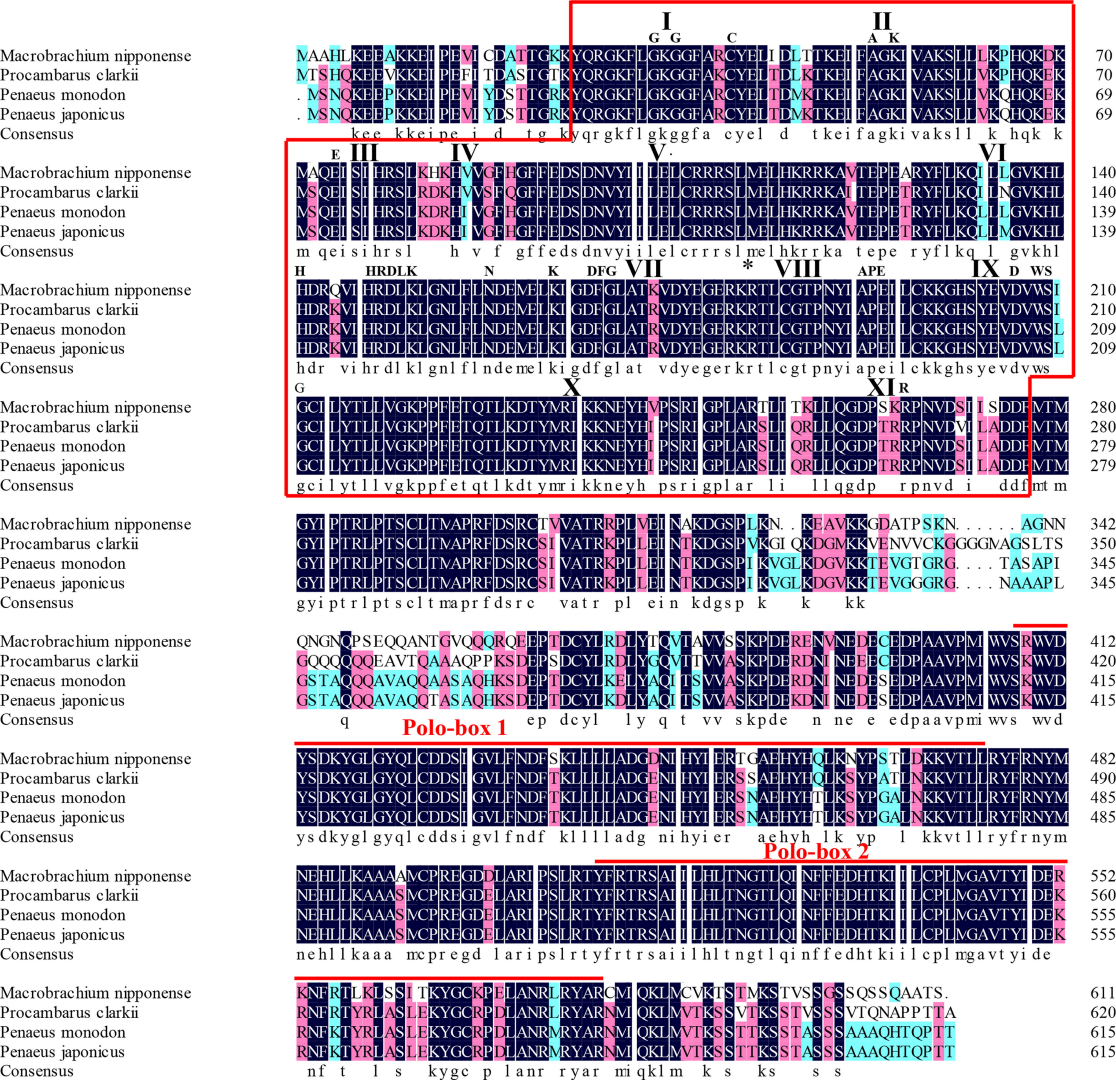
The similarity identity of amino acid sequences of *Plk1* between different species. The Serine/Threonine (Ser/Thr) kinase domain composed of sub-domains I to XI is boxed. Important motifs and residues conserved in Ser/Thr kinases (HRDLKxN, KxxDFG, APE, DxWSxG and R) are indicated above the alignment. The asterisk indicates the position of the conserved T182 residue essential for kinase activation and the bullet point indicates the L104 residue involved in BI 2536 binding. The two Polo-box domains (PBD) are over-lined.

The protein sequence of *Mn-Plk1* shared over 80% identity with the *Plk1* sequences from other crustacean species in NCBI based on the BLASTx analysis. The species included *Procambarus clarkii* (83.03%, XP_045623220.1), *Penaeus monodon* (82.42%, XP_037785985.1), *Penaeus chinensis* (82.42%, XP_047493190.1), *Penaeus japonicus* (82.06%, XP_042864001.1), and *Penaeus vannamei* (81.20%, XP_027211725.1) (**Figure 2**). Protein sequence alignment of *Plk1* from different species revealed that *Mn-Plk1* contains the common structure of Polo members, including a highly conserved N-terminal kinase and C-terminal protein binding domain (PBD). The conserved N-terminal kinase is composed of a serine/threonine kinase domain, of which the sub-domains I to XI have been labelled (**Figure 2**). The C-terminal PBD is composed of two Polo-boxes called Polo-box 1 and Polo-box 2 (**Figure 2**).

### Phylogenetic tree analysis

**Figure 3:**
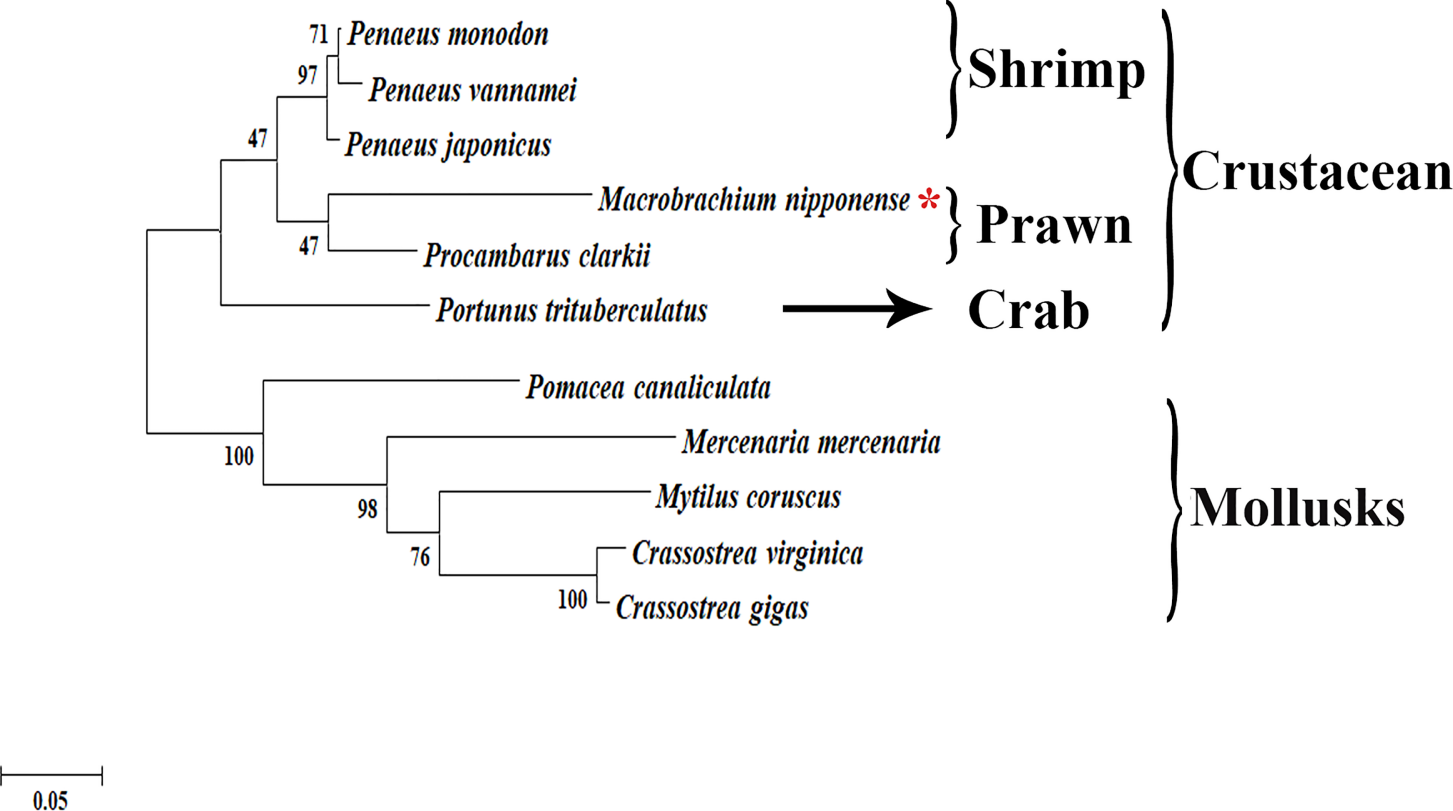
The phylogenetic tree of *Plk1* from different organisms based on amino acid sequence comparisons. Species names of *Plk1* are listed on the right of the tree. Red asterisk indicated *M. nipponense*.

The phylogenetic tree analysis revealed that the protein sequences of *Plk1* from different species were mainly divided into two branches: crustacean species and mollusks species (**Figure 3**). *Mn-Plk1* was clustered into the crustacean branch and had the closest evolutionary distance with that of crayfish, and then it clustered with the *Plk1*s of marine shrimp, followed by that of crabs. The evolutionary distance of *Mn-Plk1* with *Plk1*s of mollusks species was much more distant.

### qPCR analysis

**Figure 4:**
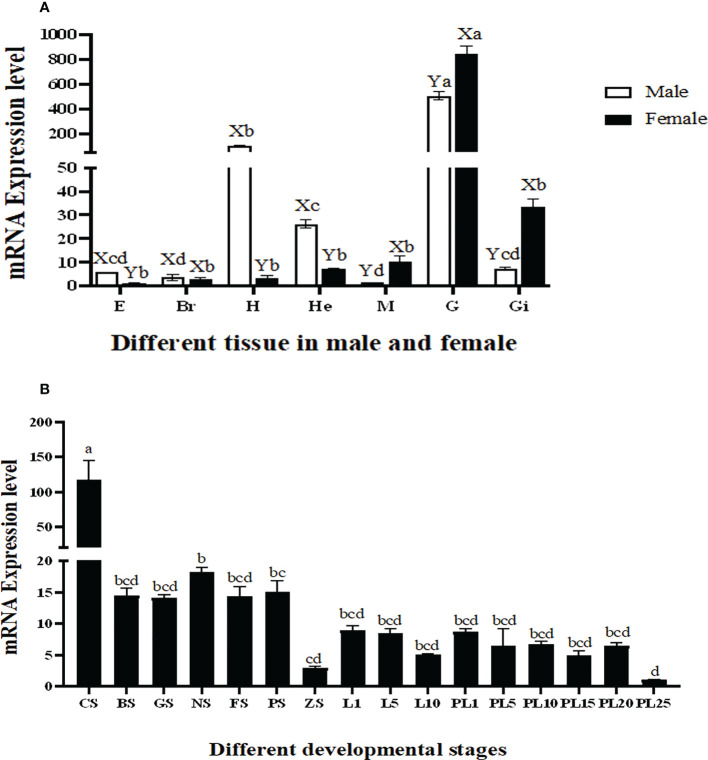
Measurement of the *Mn-Plk1* expression in different mature tissues and developmental stages by qPCR. *The amount of Mn-Plk1 mRNA was normalized to the EIF transcript level. Data are shown as mean ± SD (standard deviation) of tissues from three separate individuals. Lowercases* (a-d) *on the bars indicate expression differences among all tissues from both sex (ANOVA). Capital letters (X, Y) on the bars indicate the expression differences in the same tissue between male and female prawns (independent sample T-test).* **(A)** *Mn-Plk1* expression in different mature tissues. **(B)** *Mn-Plk1* expression in different developmental stages. E, eyestalk; Br, brain; H, heart; He, hepatopancreas; M, muscle; G, gonad; Gi, gill; CS, cleavage stage; BS, blastula stage, GS, gastrula stage; NS, nauplius stage; FS, posterior nauplius stage; PS, protozoea stage; GS, zoea stage; L, larval developmental stage; PL, post-larval developmental stage.

The qPCR analysis revealed that the highest expression of *Mn-Plk1* occurred in the testis and ovary of male and female prawns, respectively, and it was significantly higher than the expression in the other tested tissues (*P* < 0.01). The expression levels in the testis and ovary were 505.56-fold and 840.39-fold higher than that of the female eyestalk, which had the lowest expression of all of the tested tissues. *Mn-Plk1* expression levels were higher in the eyestalk, brain, heart, and hepatopancreas of male prawns than of female prawns, whereas the expression levels in the muscle, gonad, and gill showed the opposite pattern (**Figure 4A**).

*Mn-Plk1* expression was widely detected during the whole developmental process of juvenile prawns, but peak expression occurred at the cleavage stage and was significantly higher than that at the other tested stages (*P* < 0.01). The expression levels during the larval and post-larval developmental stages (PL) generally remained stable and showed no significant differences (*P* > 0.05) (**Figure 4B**). The lowest expression of *Mn-Plk1* was observed at PL25, and the value at cleavage stage was 117.57 folds higher than that of PL25.

**Figure 5:**
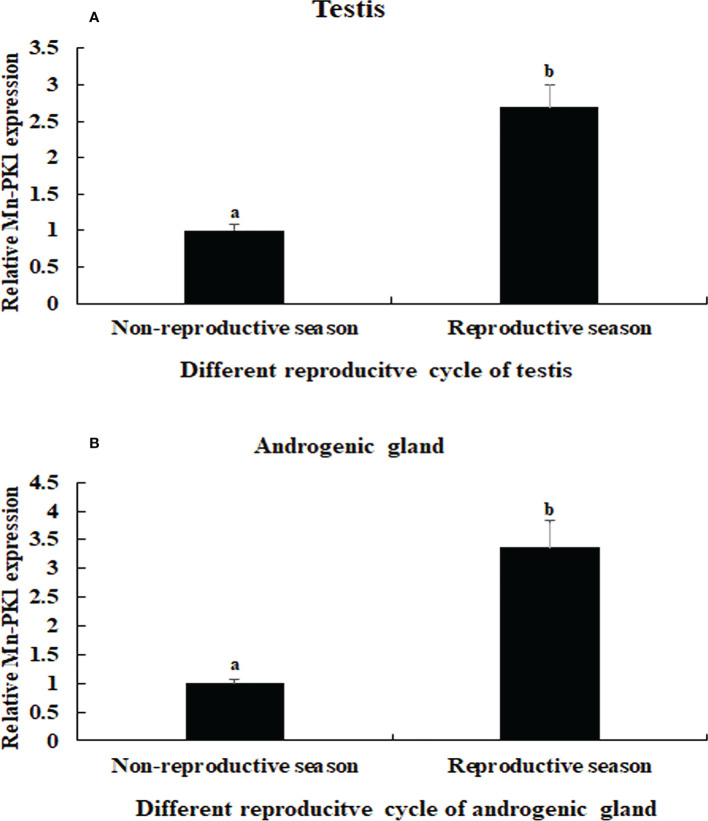
Measurement of the *Mn-Plk1* expression in the testis and androgenic gland taken from different reproductive season. *The amount of Mn-Plk1 mRNA was normalized to the EIF transcript level. Data are shown as mean ± SD (standard* deviation*) of tissues from three separate individuals. Lowercases* (a, b) *on the bars indicate expression differences between different samples (independent sample T-test).* **(A)** *Mn-Plk1* expression in the testis taken from different reproductive season. **(B)** *Mn-Plk1* expression in the androgenic gland taken from different reproductive season.

The qPCR analysis also revealed that *Mn-Plk1* expression was higher in the testis and androgenic gland from samples collected during the reproductive season than during the non-reproductive season. The values in the testis and androgenic gland from the reproductive season were 2.69-fold and 3.36-fold higher than those from the non-reproductive season, respectively, and the differences were statistically significant (*P* < 0.01) (**Figure 5**).

### RNAi analysis

**Figure 6:**
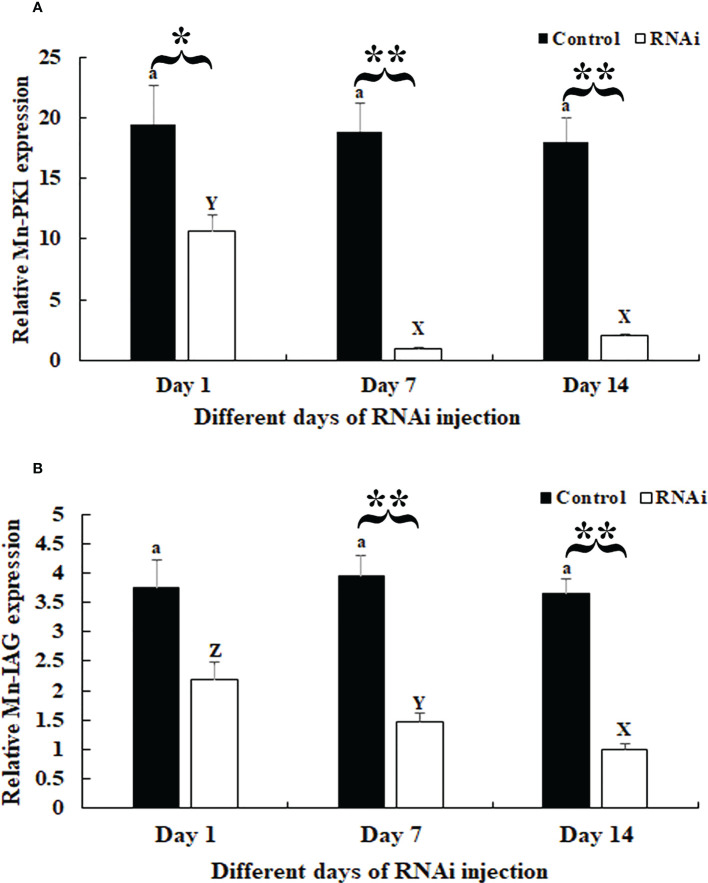
Measurement of *Mn-Plk1* and *Mn-IAG* expression at different days after *dsPlk1* and *dsGFP* injection*. The amount of Mn-Plk1 and Mn-IAG mRNA was normalized to the EIF transcript level. Data are shown as mean ± SD (standard deviation) of tissues from three separate individuals. Lowercases (a and b) on the bars indicate the expression differences between different days in the dsGFP* injected prawns, while capital letters (X, Y and Z) *on the bars indicate expression differences between different days in the dsPlk1* prawns (ANOVA). *(P < 0.05) and **(P *< 0.01) indicates significant expression difference between the RNAi group and control group at the sample day (independent sample T-test).* **(A)** *Measurement* of *Mn-Plk1* expression at different days after *dsGFP* and *dsPlk1* injection. **(B)** *Measurement* of *Mn*-*IAG* expression at different days after *dsGFP* and *dsPlk1* injection.

In the RNAi experiment, qPCR analysis revealed that the *Mn-Plk1* expression remained at a stable level in the androgenic gland after the injection of *dsGFP* (*P* > 0.05), whereas it was significantly decreased after the injection of *dsPlk1*. The decrease reached 94.8% and 88.9% at days 7 and 14, respectively, after the injection of *dsPlk1* compared with the injection of *dsGFP*, and the values were significantly different (*P* < 0.01) (**Figure 6A**).

The expression of *Mn-IAG* in the same cDNA templates also remained at a stable level in the *dsGFP*-injected prawns (*P* > 0.05), but it decreased in the *dsPlk*-injected prawns. Decreases of > 55% and 60% were observed at days 7 and 14, respectively, in the *dsPlk1* group compared to the *dsGFP* group (*P* < 0.01) (**Figure 6B**).

**Figure 7:**
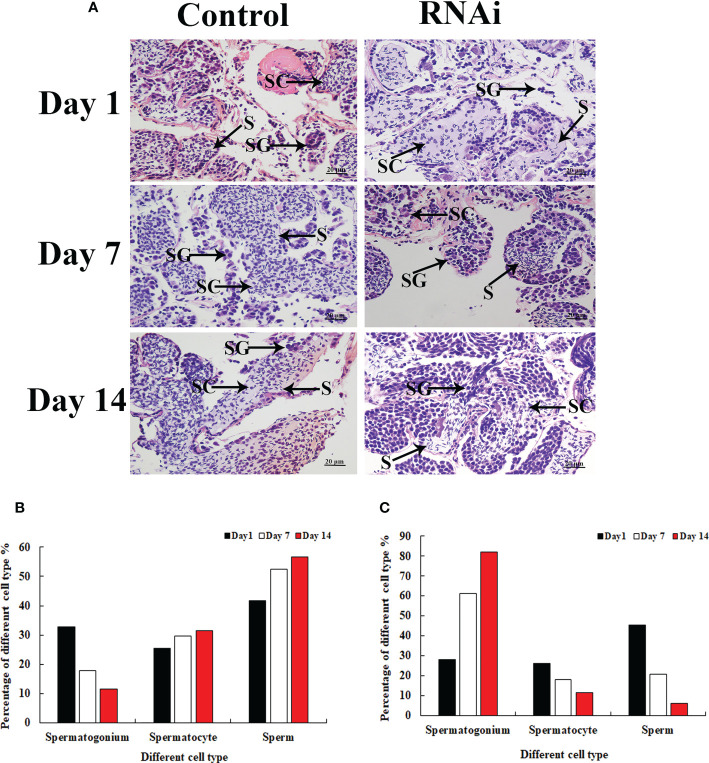
*The histological observations of testis between RNAi and control group.* SG, Spermatogonia; SC, spermatocyte; S, sperm. Scale bars = 20 μm. **(A)** *The histological observations of testis taken from the* dsGFP *and* dsPlk1 *injected prawns.* **(B)** *The percentage of different cell types in the testis of* dsGFP *injected prawns.* **(C)** *The percentage of different cell types in the testis of* dsPlk1 *injected prawns.*.

Histological observation revealed significant morphological differences between the testis from *dsGFP*- and *dsPlk1*-injected prawns. The cells in the testis were almost the same on different days after *dsGFP* injection, and sperm were the dominant cells, which were dramatically more than that of spermatogonia and spermatocyte (**Figures 7A, B**). However, the number of sperm decreased with time after *dsPlk1* injection, while the percentages of spermatogonia and spermatocytes increased. Sperm (approximate 5%) were rarely observed on day 14 in *dsPlk1*-injected prawns, while over 80% cells were observed as spermatogonia (**Figures 7A, C**).

## Discussion

*Plk1* plays vital roles in the regulation of maturation of centrosomes during the G2/M transition, the separation of centrosomes, and the activation of cyclin-dependent kinase 1 expression and spindle assembly during the cell cycle (26, 27). *Plk1* was tested in therapies for primary colorectal cancer (29), prostate and pancreatic cancers (30), and rectal cancer (31). However, studies of the role of *Plk1* in gonad development are scarce. A previous study predicted that *Plk1* has potential functions in the reproductive development of male *M. nipponense* (14). In the present study, we investigated the regulatory functions of *Plk1* in testis development of *M. nipponense* using qPCR, RNAi analysis, and histological observations.

BLASTx analysis revealed that the protein sequence of *Mn-Plk1* shared over 80% identity with the *Plk1* protein sequences from other crustacean species. Furthermore, protein sequence alignment analysis of *Plk1* identified some common structures of Polo members, including a highly conserved N-terminal kinase with a serine/threonine kinase domain and a C-terminal PBD with two Polo-boxes, which was consistent with a previous report (33). These results indicated that the cDNA sequence obtained in this study was the *Plk1* sequence of *M. nipponense*. The phylogenetic tree analysis showed a close evolutionary distance between *Mn-Plk1* and the *Plk1*s of other crustacean species but a more distant relationship with those of mollusks species. *Mn-Plk1* has the closest evolutionary distance with the *Plk1* of *P. clarkii*, which is the only freshwater prawn with a *Plk1* sequence in NCBI. Further studies should identify more *Plk1* sequences from freshwater prawns in order to promote the evolutionary analysis of *Mn-Plk1*.

In humans, overexpression of *Plk1* can promote mitosis and normal bipolar spindle assembly, while inhibition the *Plk1* expression delays mitosis (47). However, no study to date has reported qPCR analysis of *Plk1* in any aquatic species. In the present study, the highest expression of *Mn-Plk1* mRNA occurred in the testis and ovary of male and female prawns, respectively, and it was significantly higher than expression levels in the other tested tissues (*P* < 0.01). This result suggests that *Plk1* plays essential roles in the regulation of gonad development in *M. nipponense* and that the process of mitosis is much vigorous in the gonad than in the other tissues. In addition, *Mn-Plk1* expression levels in the testis and androgenic gland collected during the reproductive season were 2.69-fold and 3.36-fold higher than those taken during the non-reproductive season, respectively. Previous studies identified significant morphological differences in these organs between the reproductive and non-reproductive seasons, with much greater development during the former than the latter (48, 49). These results indicate that *Plk1* is a strong candidate gene for involvement in the reproductive development of male *M. nipponense*. Our data showed that the peak expression of *Plk1* occurred at the cleavage stage, indicating the process of mitosis was strongest during this stage.

Knockdown of the expression of *Plk1* by RNAi has been shown to inhibit the proliferation of pancreatic cancer (50) and colorectal cancer (51) cells. RNAi has also been widely used to identify the functions of male reproductive genes in *M. nipponense* (52, 53). In the present study, a > 85% decrease of *Mn-Plk1* expression was observed in the androgenic gland of *dsPlk1*-injected prawns on days 7 and 14 compared to that of *dsGFP*-injected prawns, indicating that the synthesized *dsPlk1* in the present study efficiently knocked down the expression of *Plk1* in *M. nipponense*. Interestingly, the decreased *Mn-Plk1* expression led to a significant decrease of *Mn-IAG* expression, indicating that *Plk1* positively regulates *IAG* expression in *M. nipponense*. *IAG* has been proven to promote male sex differentiation and reproduction in crustacean species, especially testis development (16–18). Histological observation revealed morphological differences between the testis of *dsPlk1*- and *dsGFP*-injected prawns. For example, on day 14 approximate 5% of cells were identified as sperm in the prawns injected with *dsPlk1*, and this number was much lower than that in *dsGFP*-injected prawns. This result indicated that *dsPlk1* had a significant inhibitory effect on testis development in *M. nipponense*.

In conclusion, our results indicate that *Plk1* regulates testis development by affecting *IAG* expression in *M. nipponense*. These results provide valuable information that can be applied to establish artificial techniques to regulate testis development in this species through editing the gene sequence of *Mn-Plk1* by CRISPR or knockdown the expression levels of *Mn-Plk1* during the sex-differentiation sensitive period by RNAi.

## Data availability statement

The original contributions presented in the study are included in the article/supplementary material. Further inquiries can be directed to the corresponding authors.

## Ethics statement

Permission to conduct the experiments was obtained from the the Freshwater Fisheries Research Center (2021JBFM02, 12-06-2021). All experiments were performed in accordance with Jiangsu Laboratory’s Animal Management Guidelines (014000319/2008-00079). Written informed consent was obtained from the owners for the participation of their animals in this study.

## Author contributions

ShJ wrote the manuscript. PW performed the RACE cloning, WZ analyzed the sequence characteristics. SuJ provided the experimental prawns. HQ performed the qPCR analysis. YG performed the RNAi analysis. YW performed the histological observations. YX revised the manuscript. HF supervised the manuscript. All authors contributed to the article and approved the submitted version.

## Funding

This research was supported by grants from the Natural Science Foundation of Jiangsu Province (BK20221207), Central Public-Interest Scientific Institution Basal Research Fund, CAFS (2021JBFM02 and 2020TD36), National Key R&D Program of China (2018YFD0900201), Jiangsu Agricultural Industry Technology System (JATS[2020]461), China Agriculture Research System-48 (CARS-48), and New Cultivar Breeding Major Project of Jiangsu Province (PZCZ201745).

## Conflict of interest

The authors declare that the research was conducted in the absence of any commercial or financial relationships that could be construed as a potential conflict of interest.

## Publisher’s note

All claims expressed in this article are solely those of the authors and do not necessarily represent those of their affiliated organizations, or those of the publisher, the editors and the reviewers. Any product that may be evaluated in this article, or claim that may be made by its manufacturer, is not guaranteed or endorsed by the publisher.
